# The pro-inflammatory potential of T cells in juvenile-onset systemic lupus erythematosus

**DOI:** 10.1186/1546-0096-12-4

**Published:** 2014-01-16

**Authors:** Lucy E Ballantine, Joanne Ong, Angela Midgley, Louise Watson, Brian F Flanagan, Michael W Beresford

**Affiliations:** 1 Department of Women’s and Children’s Health, Institute of Translational Medicine, University of Liverpool, Alder Hey Children’s NHS Foundation Trust Hospital Eaton Road, Liverpool L12 2AP, UK; 2 Department of Paediatric Rheumatology, Alder Hey Children’s NHS Foundation Trust Hospital Eaton Road, Liverpool L12 2AP, UK

**Keywords:** Juvenile-onset systemic lupus erythematosus, Lupus, IL-17A, Th17

## Abstract

**Background:**

T cells are important to systemic lupus erythematosus (SLE) disease progression. This study determined the pro-inflammatory potential of T cells within the rare condition juvenile-onset SLE (JSLE).

**Method:**

IL-17A and Th1/Th2-related cytokine concentrations were measured in plasma/serum from JSLE patients (n = 19, n = 11) and HC (n = 18, n = 7). IL17A, RORC, IL23 and IL23R mRNA were measured in peripheral blood mononuclear cells (PBMCs) from JSLE and healthy controls (HC) (n = 12). Th17-associated cytokine expression was analysed in the supernatant of CD3/CD28 activated JSLE (n = 7) and HC (n = 6) PBMCs.

**Results:**

JSLE plasma IL-17A level (21.5 ± 5.2 pg/ml) was higher compared to HC (7.2 ± 2.5 pg/ml, p = 0.028). No differences were found in Th1/Th2 cytokines levels. IL = 17A (p = 0.022), IL-6 (p = 0.028) and IL-21 (p = 0.003) concentrations were increased in supernatants from activated JSLE PBMCs. IL-17 F (p = 0.50) and IL-22 (p = 0.43) were also increased but were not statistically significant. IL17A and IL23 mRNA was significantly higher in JSLE PBMCs (p = 0.018 and p = 0.01).

**Conclusion:**

JSLE T cells have an increased ability to secrete Th17 associated cytokines once activated, which could contribute to the pro-inflammatory disease phenotype seen in these patients.

## Background

The autoimmune disease Systemic Lupus Erythematosus (SLE) is characterised by chronic inflammation, autoantibody production and severe organ damage. Juvenile-onset SLE (JSLE) is less common than adult onset disease affecting between 6 and 30 people per 100,000 depending on ethic group [1]. JSLE manifests before 17 years of age, presents with significantly more active disease, and is associated with greater tissue damage and increased patient mortality in comparison to adult-onset lupus [2]. There is no cure and most treatments focus on reducing tissue inflammation. Better understanding of the inflammatory pathways resulting in JSLE is essential for the development of improved treatment strategies for these patient groups.

Evidence, primarily from studies of adult-onset disease, indicates SLE development is characterised by a critical imbalance in T lymphocyte homeostasis, resulting in dysregulated or abnormal cytokine expression and autoantibody production [3-6]. The main T cell subsets which are central for T cell homeostasis are CD4^+^ T helper (Th) cells and regulatory T cells (Tregs) [7].

Within SLE, CD4^+^ T cells are known to regulate B cell autoantibody production through provision of co-stimulatory signals and cytokines [8]. However the role of individual T cell subsets in JSLE whilst central to disease progression remains poorly defined. Early studies of adult-onset SLE hypothesised a single CD4^+^ T cell subset was a key mediator of disease [3,9], focussing on the T helper (Th) 1 or Th2 subtypes characterised by expression of interferon gamma (IFNɣ) or Interleukin (IL) 4 respectively. More recently the identification of Th17 cells characterised by the production of IL-17A, IL-17 F, IL-21 and IL-22 and the expression of the IL-23 receptor (IL-23R) has led to the suggestion of other T cell subsets contributing to disease progression. Alternatively, an imbalance of T cell subsets which cross regulate each other or disproportionate Th17-to-Treg ratio may contribute to the pro-inflammatory profile observed in adult-onset disease [4-6,10-13]. The pathogenesis of juvenile-onset lupus may depend on the predominance of specific effector T-cell subsets leading to tissue inflammation. This study aimed to analyse the pro-inflammatory potential of T cell populations, particularly with regard to Th17 cells.

## Methods

### Patients and controls

The Liverpool Paediatric Research Ethics Committee approved this study. Written informed consent was obtained from all participating subjects or their parent/guardian. JSLE patients fulfilled the revised American College of Rheumatology (ACR) criteria for the diagnosis of SLE [14] before the age of 17 years. Paediatric non-inflammatory controls were children without inter-current infection or non-inflammatory musculoskeletal symptoms and had undergone elective surgery. All paediatric patients were recruited at Alder Hey Children’s National Health Service (NHS) Foundation Trust. JSLE disease activity data was collected using a paediatric adaptation of the British Isles Lupus Assessment Group (BILAG) 2004 (pBILAG2004) disease activity score [15].

### Cell preparation

Peripheral blood mononuclear cells (PBMCs) were isolated from fresh heparinised blood within 1 hour of collection by 1-step centrifugation through Polymorph Prep (Axis-shield, UK) following manufacturer’s instructions.

### CD3/CD28 PBMC activation and measurement of cytokines

PBMCs were re-suspended at a concentration of 1 × 10^6^ cells/ml in RPMI 1640 medium (Sigma-Aldrich) supplemented with 10% fetal calf serum (Sigma-Aldrich) and 1% penicillin/Streptomycin (Sigma-Aldrich). PBMCs were seeded into a 96 well culture plate (Nunc) at 5 × 10^5^/well in triplicate and stimulated with beads coated with antibodies to CD3/CD28 (2.5 × 10^5^ beads/well; Miltenyi Biotech) producing a final bead-to-cell ratio of 1:2. The PBMC were incubated at 37°C in an atmosphere containing 5% CO_2_. After 2 days cell free supernatants were collected and the concentration of IL-17A in supernatants was assessed using an enzyme-linked immunosorbant assay (ELISA) kit according to the manufacturer’s instructions (ebioscience, San Diego, US), the detection limit was 7.8 pg/ml.

### Measurement of cytokine concentrations

IL-17A concentration in plasma samples was measured using a commercially available Human IL-17A Immunoassay Quantikine® ELISA kit (R + D Systems Inc., Minneapolis, MN, USA) following manufacturer’s instructions.

Levels of IL-6, IL-21, IL-22 and IL-17 F in cell culture supernatant were measured using Bio-plex Pro Human Th17 cytokine assay and levels of IL-12, IFNγ, IL-4 and IL-13 were measured in serum samples using a Bio-Plex Pro Human Cytokine 27-Plex Panel (Bio-Rad Laboratories) following the manufacturer’s instructions.

### mRNA preparation and qRT-PCR

RNA was extracted from PBMCs using the RNeasy Mini kit (Qiagen Inc.). The concentration and purity of RNA was confirmed by the relative absorbance at 260 nm and measuring the 260/280 nm ratio using an ND-1000 NanoDrop spectrometer (Thermo Scientific). First strand cDNA synthesis was initiated from 100 ng total RNA using random hexamers (Promega) and avian myeloblastosis virus reverse transcriptase (Promega) using conditions described by the manufacturer in a final volume of 25 μl. The primers used were as follows: IL17A forward 5′ GAA TCT CCA CCG CAA TGA GGA CCC 3′; reverse 5′ GTT GAT GCA GCC CAA GTG GCG 3′; RORC forward 5′ GTC CCG AGA TGC TGT CAA GT 3′; reverse 5′ TGA GGG TAT CTG CTC CTT GG 3′; IL23R forward 5′ ACA GGG CAC CTT ACT TCT GAC AA 3′; reverse 5′ AGC AAA GAC GAT CAT TCC CAA T 3′; IL23 forward 5′ AGA GGG CAC CTT ACT TCT GAC AA 3′; reverse 5′ AGC AAA GAC GAT CAT TCC CAA T 3′; RPL13A forward 5′ TTT CCA AGC GGC TGA CGA AG 3′; reverse 5′ AGC AAA GAC GAT CAT TCC CAA T 3′. All quantitative real-time PCR took place using the SYBR green fluorescence method with SYBR green qPCR mastermix (Stratagene) as specified by the manufacturer. Real-time PCR reactions took place in triplicate on a MX4000® Multiplex Quantitative QPCR system (Stratagene) using standard thermal cycling conditions. Non-template controls were prepared by replacing the cDNA fraction of the PCR reaction with an equivalent volume of nuclease free water (Promega). RPL13A was monitored as an internal standard for the PBMCs. mRNA expression for each gene was normalised to these internal standards. Absolute quantification was obtained using the standard curve method of analysis.

### Statistical analysis

All data are presented as the mean ± SEM. Comparisons between JSLE and control patients were made using the Mann–Whitney test. All analyses were performed using GraphPad Prism 4 software (Graph Pad Software, San Diego, CA). p < 0.05 were considered significant.

## Results

### Clinical characteristics of patients

Thirty JSLE patients were studied; Table 1 presents data for the demographic, clinical biomarker of disease activity, physician’s global assessment of disease activity, pBILAG2004 disease activity index and current medications of the patient group. Disease activity at time of sampling varied from mild to severe, with a range of therapeutic modalities being used. Due to small volumes of blood obtained from the paediatric samples, we were unable to use the same group of JSLE patients for every experiment. Additional file 1: Table S1–S4 describe patient subsets used in each part of this study.

**Table 1:** Juvenile-onset SLE patient data

**Demographics**	**Juvenile-onset SLE patients (n = 30)**
Number (%) female	22 (73%)
Ethnicity, number	
White British	22
White other	1
Asian	7
Age at sampling, mean (range) years	12.4 (3.4 – 17.9)
Disease duration, mean (range) years	2.9 (0.01 – 10.2)
Biomarker/disease activity parameter, mean (range)	
ESR, mm/hour (normal 2 – 8 mm/hour)	9.3 (<1 – 60)
CRP, mg/litre (normal 0 – 8 mg/litre)	6.8 (<4 – 78)
C3, gm/litre (normal 1.1 – 1.9)	1.03 (0.4 – 1.37)
C4, gm/litre (normal 0.19 – 0.56)	0.20 (0.08 – 0.46)
Anti-dsDNA titre, IU/ml (normal <7)	7.6 (0 – 96)
C-HAQ score, 0–3, mean (range)	0.32 (0 – 1.5)
Physician’s global assessment of disease activity by VAS, mean (range) mm	20 (0 – 75)
SLEDAI score, mean (range)	4.4 (0 – 17)
BILAG-2004	
Number with grade A or grade B	9
Score, mean (range)	2.03 (0 – 8)
Current medications number of patients	
Hydroxychloroquine	23
Methotrexate	3
Azathioprine	7
Mycophenolate mofetil	12
Prednisolone	13
Prednisolone dosage, mean (range) mg/day	12.2 (1 – 40)
Rituximab	4

Data on demographics, biomarker parameters, pBILAG2004 disease activity scores, and current medications in patients with juvenile-onset SLE.

BILAG = British Isles Lupus Assessment Group; SLE = systemic lupus erythematosus; ESR = erythrocyte sedimentation rate; CRP = C-reactive protein; anti-dsDNA = anti–double-stranded DNA; C-HAQ = Childhood Health Assessment Questionnaire; VAS = visual analog scale (0–100-mm).

Fifty-seven paediatric non-inflammatory healthy patients were included as controls mean age 13.1 years (range 2–17 years); of which 27 were female (47%). All were of white British ethnicity.

### JSLE serum and plasma has a bias toward Th17 cytokines compared to healthy control

**Figure 1:**
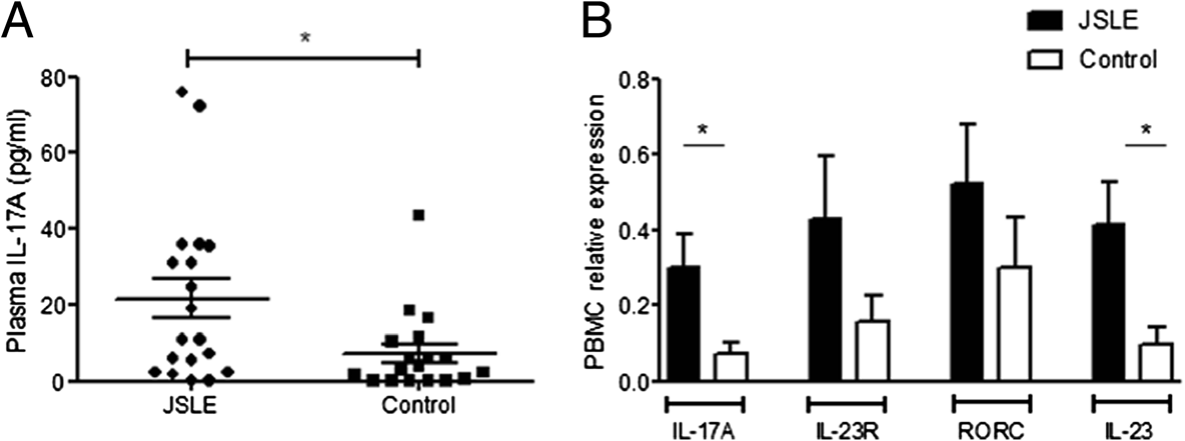
**Cytokines measurement in JSLE patient plasma and serum. (A)** IL-17A was measured in plasma from 19 JSLE patients (n = 19; BILAG -2004, A/B = 10; Score, mean (range) 3.1 (0–11)) and 18 healthy controls. JSLE patient levels were significantly higher IL-17A plasma levels compared to healthy controls p = 0.028. **(B)** PBMCs were isolated from JSLE (n = 12; BILAG -2004, A/B = 6; Score, mean (range) 2.9 (0–8)) and control (n = 12) patients, all genes are expressed relative to the housekeeping gene RPL13A. IL17A, IL23R, RORC and IL23 mRNA were higher in JSLE PBMCs compared to control with IL17A and IL23 mRNA level reaching statistical significance (p = 0.018 and p = 0.01). Data are shown as the mean ± SEM and a Mann–Whitney U test was performed to determine significance values.

To determine which T helper cell phenotype are active *in vivo* during ongoing JSLE disease, cytokine levels in the blood were measured (Figure 1). JSLE patients (n = 19) were found to have a significantly higher level of IL-17A (21.5 ± 5.2 pg/ml) in comparison to controls (n = 18, 7.2 ± 2.5 pg/ml, p = 0.028; Figure 1A). mRNA levels of IL-17A, RORC (gene encoding Th17 transcription factor (RORɣT), the Th17 stabilising cytokine IL-23 and its receptor IL-23R were measured in JSLE and control PBMCs (Figure 1B). The relative expression of IL-17A and IL-23 were both significantly higher in JSLE PBMCs in comparison to those of healthy control patients (p = 0.018 and p = 0.010 respectively). The levels of RORC and IL-23R were also higher in JSLE PBMCs but did not reach statistical significance (Figure 1B).

Levels of the Th1 cytokines IL-12 and IFNγ and the Th2 cytokines IL-4 and IL-13 were not statistically significantly increased in JSLE patients (n = 11); BILAG -2004, A/B =6 score, mean (range) (3.8 (0–12)) compared to control (n = 7) patient serum (data not shown). These data indicate that Th17 cell may be the dominant Th cell in JSLE patients, reflected by the significantly higher levels of IL-17A in patient plasma.

Comparison of JSLE patients who had active renal disease (pBILAG2004 score of A or B; n = 6) to those who did not (pBILAG2004 score C-E; n = 13) demonstrated that patients with active renal disease at the time of sampling had higher plasma levels of IL-17A but this difference was not significant (23.92 ± 4.5 pg/ml versus 17.46 ± 6.1 pg/ml, p = 0.16). However, it should be noted that renal JSLE patients had statistically significantly higher levels of IL-17A in comparison to healthy control plasma (23.92 ± 4.5 pg/ml versus 7.23 ± 2.5 pg/ml, p = 0.005).

### Increased secretion of IL-17A and Th17 related cytokines by PBMCs from JSLE patients stimulated with CD3/CD28 activation beads

**Figure 2:**
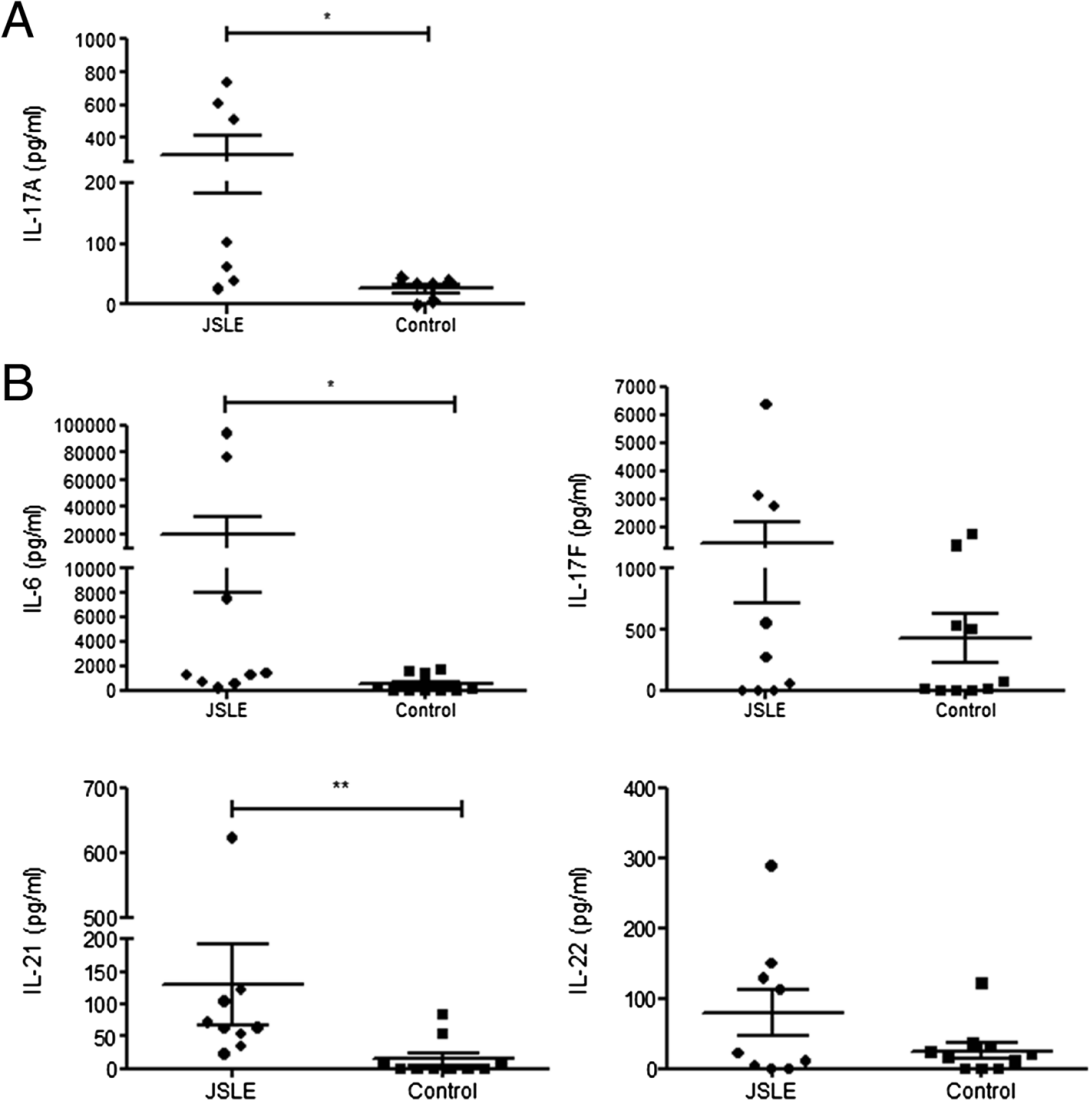
**IL-17A and Th17 associated cytokine measurement of the cell culture supernatant from CD3/CD28 activated PBMCs. (A)** PBMCs from JSLE (n = 7) and control (n = 6) patients were stimulated for 2 days using CD3/CD28 activation beads and IL-17A levels measured in culture supernatants. JSLE patients had a significantly increased level of IL-17A secretion (p = 0.022). **(B)** Cell culture supernatants from JSLE (n = 9) and control (n = 10) stimulated PBMCs were analysed by bioplex for Th17 associated cytokines. The cytokine IL-6 which is associated with the generation of Th17 cells from naïve T cells was increased in JSLE PBMCs compared to controls (p = 0.028). Cytokines produced by Th17 cells such as IL-17 F (p = 0.50), IL-21 (p = 0.003) and IL-22 (p = 0.43) were also increased in JSLE PBMCs compared to controls. Data are shown as the mean ± SEM and a Mann–Whitney U test was performed to determine significance values.

To fully characterise the production of IL-17A and Th17 related cytokines, PBMCs from JSLE (n = 7) and control (n = 6) patients were stimulated with anti-CD3 and anti-CD28 coated microbeads for 2 days *in vitro* to induce T cell activation and supernatants were analysed by ELISA. Levels of IL-17A secreted from PBMCs of patients with JSLE (297.8 ± 116.3 pg/ml) were significantly higher than from control PBMCs (26.2 ± 7.3 pg.ml) (p = 0.022) (Figure 2A).

To further measure the Th17 profile of the stimulated cells, 9 JSLE PBMC and 10 control PBMC supernatants were analysed using a Th17 cytokine panel bioplex (Figure 2B). JSLE PBMCs secreted increased levels of cytokines known to be produced by Th17 cells which drive Th17 development including IL-17 F, IL-21 and IL-22 (Figure 2B); the level of IL-21 was statistically significantly higher in JSLE compared to control supernatants (p = 0.003). JSLE supernatants also contained significantly increased IL-6 (p = 0.028). These results demonstrate that upon stimulation, JSLE PBMCs have an increased ability to produce Th17 cytokines and IL-6 required for the further generation of Th17 cells.

## Discussion and conclusions

This study analysed the pro-inflammatory potential of T cell populations in JSLE patients. We have shown JSLE patients have significantly increased plasma IL-17A in comparison to healthy controls but no differences in serum levels of Th1 or Th2 associated cytokines. Furthermore, *in vitro* activation of JSLE PBMCs resulted in increased IL-17A and Th17-associated cytokines (IL-17 F, IL-21, and IL-22) in comparison to control PBMCs and increased IL-6, which has been shown to be involved in Th17 differentiation.

Although CD4^+^ T cells appear to be essential in the development of lupus in mice and humans, it is still unresolved which type of Th cells plays a dominant role. Before the identification of Th17 cells, the overproduction of Th1 and Th2 cytokines including IFN-γ and IL-4 was reported in patients with SLE [16]. However, recent data from human and murine studies clearly implicates IL-17 and Th17 cells in lupus pathogenesis [4,6]. Our data show significantly elevated levels of IL-17A but not Th1 or Th2 associated cytokines suggesting that Th17 cells are the dominant active T helper cell type within our JSLE patients.

Previous studies have found that JSLE patients suffer from a higher proportion of renal disease in comparison to patients with adult-onset SLE [17]. Therefore the circulating IL-17A levels in JSLE patients who had active renal disease (pBILAG2004 score of A or B) and those who did not (pBILAG2004 score C-E) were compared. The six patients classed as having active renal disease at the time of blood sampling had higher plasma levels of IL-17A in comparison to non-active renal patients although this difference was not statistically significant. This suggests that IL-17A and Th17 cells may be more strongly associated with renal disease in our JSLE patients and are consistent with previous studies that demonstrated higher levels of IL-17A within serum of children with active renal disease [18].

Predominance of Th17 cells in JSLE was further tested by stimulation with CD3/CD28. This has the advantage of mimicking antigen dependant activation and allows cells to be stimulated *in vitro*. Our results indicate that only a proportion of the cells can be activated. This could be a reflection of the disease activity of the patients as two of the patients with higher cytokine levels had active renal disease. In addition, these results could also reflect the environment the cells have come from, as all of the JSLE patients analysed were on various forms of treatment (see Additional file 1: Table S1–S4). Medication has previously been shown to affect levels of Th17 cells and IL-17A production in rheumatoid arthritis (RA) patients [19,20].

We demonstrated increased IL-6 expression by JSLE PBMCs following CD3/CD28 stimulation which *in vivo* could support increased differentiation of Th17 cells. Cytokines known to be produced by Th17 cells including IL-17A (p = 0.022), IL-17 F, IL-21 (p = 0.003) and IL-22 were also secreted at a higher level from stimulated JSLE PBMCs in comparison to control PBMCs, indicating that JSLE PBMCs could be pre-programmed to a more pathogenic phenotype. Within the disease setting of JSLE the PBMCs may be similarly activated once they have migrated to disease affected organs. Notably, IL-17^+^ infiltrating T cells have previously been detected in the kidney’s of adult SLE patients [21] and IL-17A protein has been found in cutaneous lesions of adult SLE patients [22].

Increased serum levels of IFN-α and the IFN-α gene signature exhibited by JSLE patients are thought to significantly contribute to the development and maintenance of autoimmunity in JSLE, through the chronic activation of autoreactive T and B cells [23]. The interaction of this pro-inflammatory cytokine with IL-17 and the differentiation of Th cell subtypes warrants further investigation.

In conclusion, our data show that JSLE patient T cells have an increased ability to secrete Th-17 associated cytokines once they are co-stimulated. Further characterisation of the cells producing IL-17 in JSLE is needed. Our data provides important information for on-going research in this area. Studies that investigate the ability to reduce IL-17 cell activity are essential in order to design future therapeutic strategies.

## Abbreviations

JSLE: Juvenile onset systemic lupus erythematosus; SLE: Systemic lupus erythematosus; PBMCs: Peripheral blood mononuclear cells; Th: T helper.

## Competing interests

The authors declare that they have no competing interests.

## Authors’ contributions

LB: acquisition and interpretation of data, design of the study and drafting of the manuscript; JO: assisted in acquisition of data, AM: interpreting data and revising manuscript, LW: interpreting data and revising manuscript, BF: interpreting data and revising manuscript, MWB: design of the study, interpreting data and revising manuscript. All authors read and approved the final manuscript.

## Supplementary Material

Additional file 1: Table S1 Data for n = 19 JSLE patients included in Figure 1A (IL-17A cytokine measurement in JSLE plasma). **Table S2.** Data for n = 12 JSLE patients included in Figure 1B (mRNA analysis of JSLE PBMCs). **Table S3.** Data for n = 7 JSLE patients included in Figure 2A (IL-17A production from CD3/CD28 stimulated PBMCs). **Table S4.** Data for n = 11 JSLE patients included in Figure 2B (Th17-associated cytokine production from CD3/CD28 stimulated PBMCs). Click here for file
