# Supplementary material for: The pro-inflammatory potential of T cells in juvenile-onset systemic lupus erythematosus
Source: Pediatr Rheumatol Online J. 2014 Jan 16;12:4. doi: 10.1186/1546-0096-12-4 (PMC3898918; doi:10.1186/1546-0096-12-4)
Supplement: Additional file 1: Table S1 — Data for n = 19 JSLE patients included in Figure 1A (IL-17A cytokine measurement in JSLE plasma). Table S2. Data for n = 12 JSLE patients included in Figure 1B (mRNA analysis of JSLE PBMCs). Table S3. Data for n = 7 JSLE patients included in Figure 2A (IL-17A production from CD3/CD28 stimulated PBMCs). Table S4. Data for n = 11 JSLE patients included in Figure 2B (Th17-associated cytokine production from CD3/CD28 stimulated PBMCs). [file 1546-0096-12-4-S1.docx]

**Additional file**

**Table S1** Data for n=19 JSLE patients included in figure 1A (IL-17A cytokine measurement in JSLE plasma)

| Demographics | Juvenile-onset SLE patients (n=19) |
| --- | --- |
| Number (%) female | 12 (63%) |
| Ethnicity, number |  |
| White British | 10 |
| White other | 1 |
| African | 2 |
| Caribbean | 1 |
| Asian | 5 |
| Age at sampling, mean (range) years | 12.7 (3 - 18) |
| Biomarker/disease activity parameter, mean (range) |  |
| ESR, mm/hour (normal 2-8 mm/hour) | 14.8 (1 - 100) |
| CRP, mg/litre (normal 0-8 mg/litre) | 7.2 (<4 - 78) |
| C3, gm/litre (normal 1.1 – 1.9) | 0.9 ( 0.6 - 1.5) |
| C4, gm/litre (normal 0.19 – 0.56) | 0.18 ( 0.05 - 0.46) |
| Anti-dsDNA titre, IU/ml (normal <7) | 1.2 (0 - 23) |
| C-HAQ score, 0-3, mean (range) | 0.14 (0.12 - 1.25) |
| Physician’s global assessment of disease activity by VAS, mean (range) mm | 14.9 (1 - 50) |
| BILAG-2004 |  |
| Number with grade A or grade B | 10 |
| Score, mean (range) | 3.1 (0 - 11) |
| Current medications number of patients |  |
| Hydroxychloroquine | 16 |
| Methotrexate | 1 |
| Azathioprine | 6 |
| Mycophenolate mofetil | 11 |
| Prednisolone | 15 |
| Prednisolone dosage, mean (range) mg/day | 7.4 (3 – 15) |
| Rituximab | 1 |

**Table S2** Data for n=12 JSLE patients included in figure 1B (mRNA analysis of JSLE PBMCs)

| Demographics | Juvenile-onset SLE patients (n=12) |
| --- | --- |
| Number (%) female | 7 (64%) |
| Ethnicity, number |  |
| White British | 9 |
| White other | 1 |
| Asian | 2 |
| Age at sampling, mean (range) years | 12.4 (3 - 16) |
| Biomarker/disease activity parameter, mean (range) |  |
| ESR, mm/hour (normal 2-8 mm/hour) | 8.8 (1 - 39) |
| CRP, mg/litre (normal 0-8 mg/litre) | 4.3 (<4 - 9.9) |
| C3, gm/litre (normal 1.1 – 1.9) | 1.1 (0.6 - 1.8) |
| C4, gm/litre (normal 0.19 – 0.56) | 0.22 (0.07 - 0.74) |
| Anti-dsDNA titre, IU/ml (normal <7) | 3.5 (0 - 30) |
| C-HAQ score, 0-3, mean (range) | 0.5 (0.25 - 2.3) |
| Physician’s global assessment of disease activity by VAS, mean (range) mm | 26.8 (0 - 80) |
| BILAG-2004 |  |
| Number with grade A or grade B | 6 |
| Score, mean (range) | 2.9 (0 – 8) |
| Current medications number of patients |  |
| Hydroxychloroquine | 9 |
| Methotrexate | 2 |
| Azathioprine | 2 |
| Mycophenolate mofetil | 4 |
| Prednisolone | 6 |
| Prednisolone dosage, mean (range) mg/day | 8.2 (5 – 10) |
| Rituximab | 1 |

**Table S3** Data for n=7 JSLE patients included in figure 2A (IL-17A production from CD3/CD28 stimulated PBMCs).

| Demographics | Juvenile-onset SLE patients (n=7) |
| --- | --- |
| Number (%) female  Ethnicity, number  White British  Asian  Age at sampling, mean (range) years  Biomarker/disease activity parameter, mean (range)  ESR, mm/hour (normal 2-8 mm/hour)  CRP, mg/litre (normal 0-8 mg/litre)  C3, gm/litre (normal 1.1 – 1.9)  C4, gm/litre (normal 0.19 – 0.56)  Anti-dsDNA titre, IU/ml (normal <7)  C-HAQ score, 0-3, mean (range)  Physician’s global assessment of disease activity by VAS, mean (range) mm  BILAG-2004  Number with grade A or grade B  Score, mean (range)  Current medications number of patients  Hydroxychloroquine  Methotrexate  Azathioprine  Mycophenolate mofetil  Prednisolone  Prednisolone dosage, mean (range) mg/day  Rituximab | 6 (86%)  5  2  12 (6-16)  4.9 (3 - 10)  4.1 (4 - 4.6)  1.0 (0.6 - 1.3)  0.2 (0.07 - 0.46)  0  0.3 ( 0 - 0.7)  16.6 (6 - 55)  3  2 (0 - 6)  6  1  2  1  4  5  1 |

**Table S4** Data for n=11 JSLE patients included in figure 2B (Th17-associated cytokine production from CD3/CD28 stimulated PBMCs).

| Demographics | Juvenile-onset SLE patients (n=11) |
| --- | --- |
| Number (%) female | 9 (82%) |
| Ethnicity, number |  |
| White British | 8 |
| Asian | 3 |
| Age at sampling, mean (range) years | 12 (6 – 16) |
| Biomarker/disease activity parameter, mean (range) | 3.6 (<4 – 4.6) |
| ESR, mm/hour (normal 2-8 mm/hour) | 5.5 (1 - 16) |
| CRP, mg/litre (normal 0-8 mg/litre) | 3.6 (<4 - 4.6) |
| C3, gm/litre (normal 1.1 – 1.9) | 0.9 ( 0.6 - 1.25) |
| C4, gm/litre (normal 0.19 – 0.56) | 0.19 (0.07 -0.46) |
| Anti-dsDNA titre, IU/ml (normal <7) | 0.8 (0 - 9) |
| C-HAQ score, 0-3, mean (range) | 0.25 ( 0.63 -0.75) |
| Physician’s global assessment of disease activity by VAS, mean (range) mm | 11 (5 - 55) |
| BILAG-2004 |  |
| Number with grade A or grade B | 4 |
| Score, mean (range) | 2.1 (0 – 6) |
| Current medications number of patients |  |
| Hydroxychloroquine | 8 |
| Methotrexate | 2 |
| Azathioprine | 3 |
| Mycophenolate mofetil | 3 |
| Prednisolone | 6 |
| Prednisolone dosage, mean (range) mg/day | 6.25 (5 – 12.5) |
| Rituximab | 2 |
